# The lack of selection criteria for surgery in patients with non-colorectal non-neuroendocrine liver metastases

**DOI:** 10.1186/s12957-020-01883-y

**Published:** 2020-05-25

**Authors:** Ali Bohlok, Valerio Lucidi, Fikri Bouazza, Ali Daher, Desislava Germanova, Jean Luc Van Laethem, Alain Hendlisz, Vincent Donckier

**Affiliations:** 1 Department of Surgery, Institut Jules Bordet, Université Libre de Bruxelles, 121, Boulevard de Waterloo, 1000 Brussels, Belgium; 2 Department of Abdominal Surgery, Hôpital Erasme, Université Libre de Bruxelles, Brussels, Belgium; 3 Department of Hepato-Gastroenterology, Hôpital Erasme, Université Libre de Bruxelles, Brussels, Belgium; 4 Department of Medical Oncology, Institut Jules Bordet, Université Libre de Bruxelles, Brussels, Belgium; 5 Centre de Chirurgie Hépato-Biliaire de l’ULB (CCHB-ULB), Brussels, Belgium

**Keywords:** Non-colorectal, Non-neuroendocrine, Liver metastases, Surgery, Prognostic, Individual, Selection

## Abstract

**Background:**

The benefit of surgery in patients with non-colorectal non-neuroendocrine liver metastases (NCRNNELM) remains controversial. At the population level, several statistical prognostic factors and scores have been proposed but inconsistently verified. At the patient level, no selection criteria have been demonstrated to guide individual therapeutic decision making. We aimed to evaluate potential individual selection criteria to predict the benefit of surgery in patients undergoing treatment for NCRNNELM.

**Methods:**

Data for 114 patients undergoing surgery for NCRNNELM were reviewed. In this population, we identified an early relapse group (ER), defined as patients with unresectable recurrence < 1 year postoperatively who did not benefit from surgery (*N* = 28), and a long-term survival group (LTS), defined as patients who were recurrence-free ≥ 5 years postoperatively and benefited from surgery (*N* = 20). Clinicopathologic parameters, the Association Française de Chirurgie (AFC) score, and a modified 4-point Clinical Risk Score (mCRS) (excluding CEA level) were analyzed and compared between LTS and ER groups.

**Results:**

The majority of patients were female and a majority had an ASA score ≤ 2 at the time of liver surgery. The median age was 55 years. Almost half of the patients (46%) presented with a single-liver metastasis. Intermediate- and low-risk AFC scores represented 40% and 60% of the population, respectively. Five- and 10-year overall survival (OS) and disease-free survival (DFS) rates were 56% and 27%, and 30% and 12%, respectively. Negative prognostic factors were the size of liver metastases > 50 mm and delay between primary and NCRNNELM <24 months for OS and DFS, respectively. AFC score was not prognostic while high-risk mCRS (scores 3–4) was predictive for the poorer OS. The clinicopathologic parameters were similar in the ER and LTS groups, except the presence of N+ primary tumor, and the size of liver metastases was significantly higher in the ER group.

**Conclusion:**

In patients with resectable NCRNNELM, no predictive factors or scores were found to accurately preoperatively differentiate individual cases in whom surgery would be futile from those in whom surgery could be associated with a significant oncological benefit.

## Core tip

We reviewed a series of 114 patients who underwent surgery with curative intent for non-colorectal non-neuroendocrine liver metastases (NCRNNELM). In the whole series, a 5-year overall and disease-free survival were 56% and 30%, respectively. At the population level, none of the previously reported factors or scores was found to be prognostic. At the individual level, no baseline primary tumor or liver metastasis characteristic was able to differentiate the patients who would benefit from surgery from those who would not. These results confirm that surgery is an effective option in selected patients with NCRNNELM but strongly highlights the lack of biomarkers to guide individual therapeutic decision making.

## Introduction

In contrast to colorectal liver metastases (CRLM), the role of surgery for the treatment of patients with liver metastases from non-colorectal non-neuroendocrine origin (NCRNNELM) remains poorly defined [1]. Several reports have shown that liver resection can lead to prolonged survival and the occasional cure in these cases serve as a proof-of-concept that surgery could represent an effective therapy in a subgroup of these patients [2–4]. Different predictive and prognostic factors, mainly related to surrogate markers of primary tumor aggressiveness, including the rate of progression and the extent of metastatic disease, have been proposed [2, 3, 5]. However, apart from the fact that these factors and risk models have been inconsistently validated in external series [2, 3, 6–9], their accuracy for preoperatively distinguishing the individual patients who will or will not benefit from surgery remains undetermined. In fact, this question regarding the benefit of surgery in these oncological patients is complex as benefits may range from improved quality of life and avoidance or delay of several lines of chemotherapy to prolonged survival and cure. In patients with NCRNNELM in particular, which includes a variety of primary tumors with different tumor biologies, the results of surgery should be carefully balanced against constant improvements in systemic and locoregional treatments [5, 10]. To independently address this question in a retrospective series, it can be assumed that no oncological benefit was obtained in patients who developed rapid and unresectable postoperative recurrence, whereas true oncological benefit was obtained in patients who maintained a disease-free status for a prolonged time after surgery. Therefore, we reviewed a recent series of patients who underwent surgery for NCRNNELM and analyzed the prognostic value of several preoperative clinico-pathological factors and of the score developed by the Association Française de Chirurgie (AFC) [2]. We also tested a new simple model adapted from the clinical risk score (CRS), established for patients undergoing surgery for CRLM [11]. To evaluate the potential value of these factors as individual selection criteria for surgery, we compared the characteristics of patients defined as having obtained no benefit from surgery (i.e., the patients who developed unresectable recurrence within the first postoperative year), and patients considered to have benefited from surgery (i.e., the patients who are recurrence-free at 5 years after the first liver surgery).

## Patients and methods

### Patients

A consecutive series of patients who underwent surgery for NCNNELM between January 2005 and December 2017 were reviewed. In all cases, surgical decisions were made during multidisciplinary institutional tumor boards. When neoadjuvant treatment was administered for liver metastases, patients were considered for surgical intervention only in cases in which they responded to the treatment, as defined by stable or responding disease according to the Response Evaluation Criteria in Solid Tumor (RECIST) criteria [12]. In all patients, surgery was of curative intent for all liver metastases, consisting of radical surgical resection or clearance with radiofrequency destruction (RF). The study was approved by the Ethics Committee of Institut Jules Bordet (CE2953) and the Hôpital Erasme (P 2019/232).

### Surgery

Surgery was performed by a laparoscopic or open approach according to the surgeon’s choice. RF was preferentially reserved for centrally located lesions ≤ 30 mm. Liver resection and RF were performed under intraoperative ultrasound guidance. Resections of 3 or more liver segments were defined as major hepatectomies. Operative complications were graded according to Clavien-Dindo classification (CD), as in-hospital or within 90 postoperative days [13]. Major complications were defined as CD grade ≥ III.

### Follow-up and patient categorization

After surgery, follow-up data were collected, including clinical, laboratory, and imaging (computed tomography scan or magnetic resonance imaging) data. Two groups of patients were defined: The early relapse group (ER), including the patients who developed unresectable/non-clearable tumor recurrence in the first year after surgery, and the long-term survival group (LTS), including the patients who were disease-free at least 5 years after the first liver surgery, also including patients who underwent surgery several times for tumor recurrence. In addition, to test another cut-off that might correspond to some benefit of the surgical intervention, we also compared the ER group with the patients who were disease-free at least 3 years after the intervention.

### Analysis of potential prognostic factors and scores

Several factors, related to demographics, primary tumor type, and stage, and characteristics of liver metastases, and two scores were evaluated. First, the AFC score [2], that includes 6 factors, the origin of the primary tumor (0 to 3 points), the patient age (0 to 2 points), the disease-free interval between treatment of the primary tumor and diagnosis of the liver metastases (0 to 2 points), the presence of extrahepatic metastases (0 to 1 point), a gross residual disease resection (0 to 1 point), and the need for major hepatectomy (0 to 1 point). According to this score, patients were stratified into three groups, defined as low- (score of 1 to 3), intermediate- (score of 4 to 6), or high-risk (score > 6). Second, we used a modified CRS (mCRS) that we adapted from the traditional CRS model established for CRLM [11]. Traditional CRS includes 5 parameters, worth 1 point each, including the nodal invasion of the primary tumor (N+), a disease-free interval between the primary tumor and diagnosis of the liver metastases < 12 months, the presence of more than 1 liver metastasis, the largest liver metastasis > 50 mm, and a preoperative CEA level > 200 ng/ml. In mCRS, we considered the 4 first factors without the CEA level because it is not really relevant in NCRNNELM. Using this 4-point mCRS, patients were stratified into low- or high-risk groups according to scores of 0 to 2 and 3 to 4. The predictive values of different parameters and of the AFC and mCRS scores were evaluated in the entire series and compared between the ER and LTS groups. Similar comparisons were made between the ER group and the group of patients who were disease-free at 3 years after surgery. In addition, to evaluate the potential impact of primary tumor origin, we separately analyzed the results in patients with liver metastases from breast and non-breast origins, from digestive (including esophageal, gastric, pancreatic, small intestine) and non-digestive origins, from genitourinary and non-genitourinary origins, and from sarcoma and non-sarcoma origins.

### Statistical analysis

The data were analyzed with the statistical software SPSS v25. Descriptive analysis of all of the clinical and histological parameters was done. OS was defined as the time from the date of first liver surgery to the date of death from any cause. DFS was considered to be the time from the date of surgery to the date of detection of first recurrence or death. Survival curves were generated using the Kaplan-Meier method. For uni- and multi-variate analyses of AFC score, mCRS, and each element of both scores, hazard ratios were calculated using Cox regression analysis. Factors with univariate significance at a level of *p* value < 0.1 were entered into a multivariate Cox proportional hazards model. In multivariate models, a *p* value of < 0.05 was considered significant. Clinicopathologic parameters were compared between the ER and LTS groups using the Mann-Whitney *U* test for continuous data and with the chi-square test for categorical variables.

## Results

### Patient characteristics

Data for 114 patients undergoing surgery for NCRNNELM were analyzed. Patient demographic and clinico-pathologic characteristics are shown in Table 1. The majority of patients were female, the median age was 55 years, and the majority of patients had an ASA score ≤ 2 at the time of liver surgery. None of the patients had chronic liver dysfunction such as cirrhosis. The majority of liver metastases were of breast cancer origin (61%), followed by genitourinary, sarcoma, gastrointestinal, melanoma, and squamous cancers. None of the patients had extrahepatic metastases at the time of diagnosis and surgery for liver metastases. Lymph node metastases were present with primary tumors in 40% of the patients. In 38% and 49% of the patients, the disease-free interval between the primary tumor and liver metastases was > 12 months and > 24 months, respectively. Neoadjuvant treatment was administered for the primary tumor in 37% and adjuvant therapy in 70% of the cases. Liver metastases were multiple in 54% and > 50 mm in 20% of the cases. At the time of liver surgery, no patient had been diagnosed with extrahepatic metastases. After the diagnosis of liver metastases, 83% of patients received neoadjuvant chemotherapy before surgery and 58% received adjuvant chemotherapy. According to the AFC score, 60% had a low risk, 40% had an intermediate risk, and no patients had high-risk disease. According to mCRS, 77% had a low score and 23% had a high score.

**Table 1:** Basic characteristics of patients with NCRNNELM

Characteristics	*N* (%)
**Patients (number)**	114
**Sex: female**	90 (78%)
**Age (years), median[range], > 60 years)**	55[26–83], 37%
**BMI mean (range)**	24.6 (16.8–38.7)
**ASA 1–2**	89 (78.1%)
**Primary tumor sites**	
Breast	70 (61%)
Genito-urinary	13 (11%)
Gastro-intestinal	10 (9%)
Sarcoma	11 (10%)
Melanoma	4 (4%)
Squamous	3 (3%)
Others	3 (3%)
**Primary lymph node metastasis**	46 (40%)
**Timing of diagnosis**	
0–12 months	43 (38%)
12–24 months	13 (11%)
> 24 months	58 (51%)
**Number of nodules mean (median[range])**	1.86 [1 (1–9)]
**Multi-nodular**	62 (54%)
**Size of largest nodule mean (median[range]) (mm)**	32.85[25 (2–70)]
**Size > 50 mm**	23 (20%)
**Systemic treatment for the primary tumor**	
NACT	42 (37%)
ACT	79 (69%)
Hormonal therapy	56 (49%)
Radiotherapy	55 (48%)
Trastuzumab	25 (22%)
Glivec	9 (8%)
Other	3 (3%)
**Systemic treatment for liver metastases**	
NACT	95 (83%)
ACT	66 (58%)
**Type of surgery**	
Open approach/laparoscopy	79 (69%)/35 (31%)
RF	33 (29%)
Major hepatectomy	23 (20%)
**Complications Clavien-Dindo**	
0	91 (80%)
I–II	10 (9%)
III (a-b)	12 (11%)
IV	1 (1%)
V	0
**Relapse**	65 (57%)
Liver	37 (33%)
Liver + lung	3 (3%)
Liver + bone	3 (3%)
Liver + brain	2 (2%)
Liver + peritoneum	3 (3%)
Lung	4 (4%)
Peritoneum	3 (3%)
Bone	1 (1%)
Brain	1 (1%)
Multiple	8 (7%)
**Number of hepatectomies**	
2	11 (10%)
3	1 (1%)
**AFC score**	
1	16 (14%)
2	19 (17%)
3	33 (29%)
4	25 (22%)
5	16 (14%)
6	5 (4%)
**Low/ Intermediate risk AFC score**	68 (60%)/46 (40%)
**mCRS**	
0	24 (21%)
1	38 (33%)
2	26 (23%)
3	23 (20%)
4	3 (3%)
**Low-/high-risk (0–2)**	88 (77%)/26 (23%)

*BMI* body mass index, *ASA* American Society of Anesthesia, *LN* lymph node, *ACT* adjuvant chemotherapy, *NACT* neoadjuvant chemotherapy, *RF* radiofrequency destruction, *mCRS* modified clinical risk score

### Surgery

Surgical data and postoperative outcomes are detailed in Table 1. Surgery was mainly performed via an open approach, representing 69% of the cases. Major hepatectomies represented 20% of the cases. RF was used in 29%. Postoperative complications occurred in 20% of the cases, including major complications in 11%. No postoperative mortality was observed.

### Postoperative outcomes

**Fig. 1:**
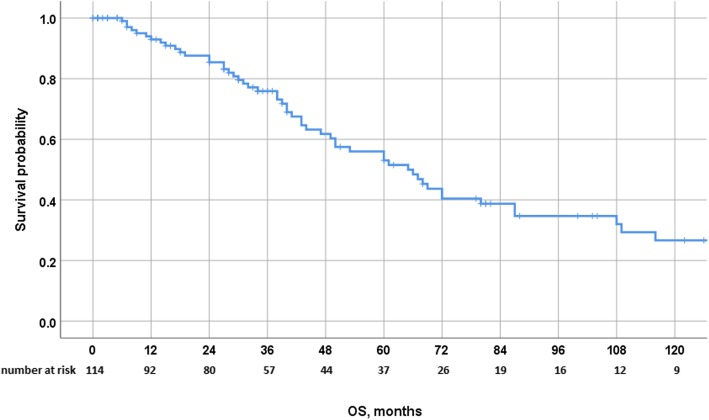
Overall survival after surgery for NCRNNELM

**Fig. 2:**
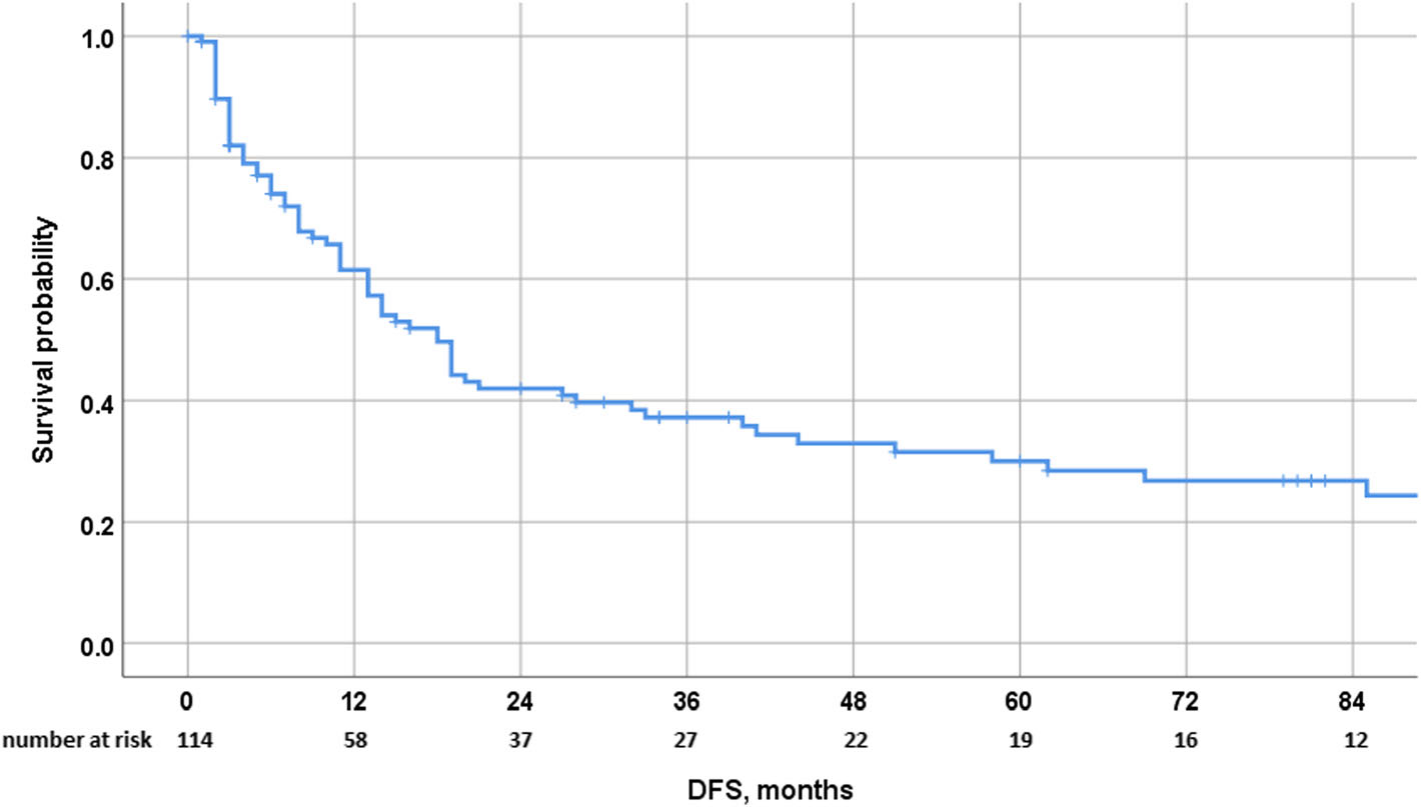
Disease-free survival after surgery for NCRNNELM

After a median follow-up of 81 months, 65 patients (57%) experienced recurrence. The median time to recurrence was 10 months. The liver represented the most common site for recurrence (44%) and the exclusive site in a third of the patients. Other sites of recurrence were the lung, peritoneum, brain, and bone in 4%, 3%, 1%, and 1%, respectively. Eleven patients (10%) underwent additional surgery for liver recurrence, including 1 patient who underwent 3 liver interventions (Table 1). In the whole cohort, 3-, 5- and 10-year OS were 76%, 56%, and 27%, respectively, with a median OS of 65 months (Fig. 1). Three- and 5-year DFS were 39% and 30%, respectively, with a median DFS of 18 months (Fig. 2). Postoperative survival was similar in patients who underwent surgery for breast cancer or for non-breast cancer liver metastases, with 3-, 5-, and 10-year OS rates of 70.2%, 50%, and 23% versus 86%, 69%, and 34%, respectively (*p* = 0.142), and 3- and 5-year DFS rates of 31% versus 23% and 49% and 42%, respectively (*p* = 0.322). Postoperative survival was also similar in patients who underwent surgery for digestive or for non-digestive cancer liver metastases, with 3- and 5-year OS rates of 81.4% and 50.9% versus 74.9% and 56.7%, respectively (*p* = 0.705), and 3- and 5-year DFS rates of 36% and 24% versus 37% and 31%, respectively (*p* = 0.966). Postoperative survival was also similar in patients who underwent surgery for sarcoma and non-sarcoma liver metastases, with 3- and 5-year OS rates of 88.9% and 71.1% versus 74.5% and 54.5%, respectively (*p* = 0.191), and 3- and 5-year DFS rates of 52.5% and 35% versus 35.7% and 29.5%, respectively (*p* = 0.4). Interestingly, 3- and 5-year OS rates in genitourinary and non-genitourinary were of 100% and 83.3% versus 73.1% and 53.3%, respectively (*p* = 0.08), and 3- and 5-year DFS rates of 75% and 75% versus 33% and 26%, respectively (*p* = 0.027) (Table 2).

**Table 2:** Survival according to liver metastasis origin

	OS			DFS		
	3 years (%)	5 years (%)	*p*	3 years (%)	5 years (%)	*p*
**Breast origin**			0.142			0.322
**Yes**	70.2	50		31	23	
**No**	86	69		49	42	
**Digestive**			0.705			0.966
**Yes**	81.4	50.9		36	24	
**No**	74.9	56.7		37	31	
**Sarcoma**			0.191			0.4
**Yes**	88.9	71.1		52.5	35	
**No**	74.5	54.5		35.7	29.5	
**Genitourinary**			0.08			0.027
**Yes**	100	83.3		75	75	
**No**	73.1	53.3		32.8	25.6	

### Univariate and multivariate regression analysis of potential prognostic factors

In univariate analysis, liver metastasis size > 50 mm and primary tumor N+ status were significantly associated with poorer OS, while only liver metastasis size > 50 mm remained predictive for poor OS in multivariate analysis (Table 3). The only significant poor predictive factor for DFS was a time interval between the diagnosis of the primary tumor and of the liver metastasis of < 24 months (*p* = 0.013). When AFC score was categorized into low and intermediate risks, no difference was observed for survival. When mCRS was categorized into low and high risks, a significant difference was observed for OS (*p* = 0.009, HR = 2.05 + 95% CI (1.12–3.76)), but not for DFS, (*p* = 0.31). The significance of mCRS for OS was not confirmed in multivariate analysis (*p* = 0.7).

**Table 3:** Univariate and multivariate analysis for OS and DFS

	OS						DFS		
	Univariate analysis			Multivariate analysis			Univariate analysis		
	HR	95% CI	*p*	HR	95% CI	*p*	HR	95% CI	*p*
**Age > 60**			0.902						0.871
**Primary location**			0.266						0.445
**Breast origin**	1.57	0.85–2.9	0.147				1.28	0.78–2.11	0.333
**Extra-hepatic metastasis**	1.47	0.78–2.77	0.231				0.94	0.53–1.67	0.839
**disease-free interval < 4 months**	1.14	0.44–2.96	0.783				2.58	1.2–5.45	0.013
**Major hepatectomy**	0.95	0.48–1.85	0.869				1.23	0.71–2.14	0.461
**AFC score Cat**	0.84	0.46–1.51	0.553				0.91	0.55–1.5	0.705
**Diam > 50 mm**	2.19	1.17–4.08	0.014	2.181	1.09–4.37	0.028	1.50	0.85–2.64	0.158
**Multi-nodular**	1.40	0.8–2.47	0.243				1.5	0.92–2.45	0.105
**Lymph node involvement**	2.08	1.18–3.64	0.011	1.987	0.93–4.23	0.075	1.28	0.78–2.1	0.331
**Disease-free interval < 12 months**	1.18	0.64–2.17	0.596				1.12	0.66–1.89	0.682
**mCRS cat**	2.05	1.12–3.76	0.02	1.123	0.48–2.61	0.787	1.31	0.75–2.29	0.352

### Comparison of the early relapse and long-term survival groups

From the overall cohort, we identified 28 patients who recurred within the first postoperative year (ER group) (24.5%) and 20 patients who were disease-free at least 5 years after surgery (LTS group) (17.5%) (Table 4). The proportions of breast NCRNNELM were similar in the 2 groups, representing 18 (46%) and 13 (65%) patients in the ER and LTS groups, respectively. Clinicopathologic parameters were similar overall between the ER and LTS groups, except for the presence of N+ primary tumor and liver metastasis size that were significantly higher in the ER group. Of note, liver metastases > 50 mm and the disease-free interval between diagnosis of the primary tumor and of the liver metastasis were not significantly differently distributed among ER and LTS patients. AFC score did not discriminate between ER and LTS patients, either for the values of the score (*p* = 0.7) or when categorized into low and intermediate risks (*p* = 0.7). Similarly, mCRS scores were similar among ER and LTS patients (*p* = 0.1), whereas low- and high-risk mCRS patients tended to be differently distributed, high-risk mCRS representing 12/28 (43%) of the ER patients and low-risk CRS 17/20 (85%) of the LTS patients (*p* = 0.059). When comparing the ER patients with those who were recurrence-free at 3 years after surgery, the ER group was associated with more lymphatic invasion at the primary tumor (*p* = 0.002), had a higher number of LMs preoperatively (*p* = 0.02), and received more neoadjuvant chemotherapy before surgery for the primary (*p* = 0.026) (Table 4). As compared with patients who were recurrence-free at 3 years, the AFC score was not significantly different in ER patients, while mCRS was significantly higher (*p* = 0.037) (Table 4).

**Table 4:** Comparison of clinicopathologic parameters between ER and LTS groups

Groups	ER (*N* = 28)	3-year LTS (*N* = 28)	*p* vs ER	5-year LTS (*N* = 20)	*p* vs ER
**Sex (female)**	22	23	1	17	0.716
**Age (years)**	50.07 [51 (30–72)]	57 [62(26–78)]	0.09	58.7 [63 (28–78)]	0.061
< 30	0	2		1	
30–60	24	11		7	
> 60	4	15		12	
**Continuous variables**	mean (median [range])			mean (Median [range])	
**BMI**	24.6 [24.2 (16.8–34.4)]	24.4 [24 (18–37)]	0.72	23.54 [23.98 (18.6–27.5)]	0.403
**ASA score**			0.861		0.859
1	1	1		1	
2	22	23		16	
3	4	4		3	
4	1	0		0	
**Type of primary**			0.226		0.396
Breast	18	17		13	
Squamous origin	0	2		2	
Melanoma	2	1		1	
Genito-urinary	2	5		3	
Sarcoma	2	3		1	
Gastro-intestinal	3	0		0	
Esophagus	1	0		0	
**Disease-free interval**			0.533		0.281
0–12 months	13	10		7	
12–24 months	2	1		0	
> 24 months	13	17		13	
**AFC score**			0.64		0.727
1	5	4		2	
2	2	4		4	
3	6	9		5	
4	9	4		4	
5	5	6		4	
6	1	1		1	
**AFC score cat**			0.422		0.770
**Low/intermediate risk**	13/15	17/11		11/ 9	
**Size of metastasis at diagnosis (mm)**	37.8 [35 (12–70)]	33 [24(10–104)]	0.058	30.8 [24 (10–100)]	0.041
**Size of metastasis pre-op (mm)**	34.18 [34 (8–65)]	29.6 [22(3–100)]	0.166	29.4 [22 (3–100)]	0.143
**Diam > 50 mm**	9	6	0.547	4	0.512
**Number of LM**	2.25 [2 (1–5)]	2 [1(1–9)]	0.104	2.25 [1 (1–9)]	1
**Number of LM pre-op**	2.07 [2 (1–4)]	1.7 [1(1–9)]	0.021	1.95 [1 (1–9)]	0.792
**Multinodular metastasis**	18	14	0.418	9	0.242
**Positive LN status of primary**	18	7	0.002	5	0.007
**Synchronous (< 12 months)**	13	10	0.135	6	0.370
**mCRS**			0.132		0.190
0	3	7		6	
1	8	10		6	
2	5	7		5	
3	9	4		3	
4	3	0		0	
**mCRS cat**			0.037		0.059
**Low-/high risk**	16 /12	24		17/ 3	
**RF**	10	5	0.371	3	0.354
**Major hepatectomy**	7	7	1	5	1
**Post-operative complications**	5	6	0.6	3	0.683
**Clavien-Dindo**			0.34		0.195
I–II	3	3		3	
IIIa	2	1		0	
**Primary**					
NACT	15	6	0.026	6	0.144
ACT	22	18	0.375	13	0.339
**Hormonal therapy**	13	15	0.79	12	0.394
**Radiotherapy**	15	14	1	11	1
**Immunotherapy**			0.449		0.676
No	19	18		13	
Trastuzumab	8	6		5	
Glivec	1	2		1	
Other	0	2		1	
**Liver metastasis**					
NACT	24	23	1	17	1
ACT	21	13	0.09	10	0.208

*RFS* relapse-free survivors, *BMI* body mass index, *ASA* American Society of Anesthesia, *LN* lymph node, *LM* liver metastases, *mCRS* modified clinical risk score, *RF* radiofrequency destruction, *ACT* adjuvant chemotherapy, *NACT* neoadjuvant chemotherapy, *ER* early relapse, *LTS* long-term survivors

## Discussion

The term NCRNNELM corresponds to a heterogeneous group of patients with liver metastases from various origins. This denomination is mainly used by surgeons to define cases where, in contrast with CRLM [14], the surgical indication remains controversial. Currently, in the absence of controlled or case-matched studies, the real benefit of surgery in patients with NCRNNELM remains difficult to assess and it is challenging to determine whether long-term postoperative survival in some of these patients results from the surgical treatment itself or from the selection of a subgroup with favorable tumor biology. However, as in patients with CRLM, long-term DFS obtained after surgery for NCRNNELM indicates that some of these patients may have an intermediate tumor progression profile, characterized by a limited metastatic capacity, as defined by oligometastatic status [15–17]. Therefore, as in CRLM, the identification of biomarkers of such oligometastatic behavior would represent highly relevant progress toward a better understanding of the biological mechanisms involved in different modes of tumor progression and for personalized therapeutic decision making. In this view, specific markers of tumor biology that can be used to predict the benefit of surgery in individual cases are critically needed, even more than population-based prognostic models. To address this question, the characterization of the long-term disease-free survivors after surgery for NCRNNELM (oligometastatic cases who benefited from surgery) as compared with the patients who developed rapid postoperative recurrences (the diffuse metastatic cases who did not benefit from surgery) may represent a first step toward identifying accurate, clinically available selection criteria. In particular, in such a heterogeneous group of patients, the respective contributions of surgery and systemic treatment for improving the outcome could be difficult to discriminate. In that sense, it might be assumed that both the patients with prolonged DFS and those with rapid recurrence after surgery represent appropriate target groups to be identified preoperatively, corresponding to the good candidates for surgery and to those in whom surgery should be contraindicated, respectively.

In this series, we observed postoperative outcomes that were similar overall to those recently reported in the literature [1, 2, 7, 18, 19]. At 3 and 5 years, OS rates were 75% and 55% and DFS rates were 40% and 30%, respectively, and surgery was associated with limited operative morbidity and no mortality, confirming that surgery may represent an effective and safe therapeutic option in selected patients with NCRNNELM [7]. Furthermore, the fact that prolonged DFS was obtained in approximately a third of the patients may serve as a proof-of-concept for the existence of oligometastatic progression in some of these cases. In contrast with other studies, we could only identify a few prognostic factors, related to the primary tumor stage and the extent of liver involvement [2, 6, 18, 19]. In multivariate analysis, only the nodal status of the primary tumor and the size of liver metastases were associated with the postoperative OS but not with the risk of recurrence. In addition, a delay of longer than 2 years between the primary and the diagnosis of liver metastases appeared to be predictive for improved DFS, but not for OS. Furthermore, the prognostic value of the model proposed by the AFC was not verified [2]. Several factors could be responsible for these discrepancies [2, 3, 7]. Primarily, the present results were obtained in highly selected patients, as indicated by the fact that almost half of them had single-liver metastases and that the patients who progressed on systemic therapy were excluded. This criterion is likely to have excluded the patients with the poorest tumor biology. Our cohort included only patients with low or moderate risk according to AFC score. Nevertheless, in the original series published by Adam, only 3% of the population had a high-risk AFC score [2], while ranging from 3 to 6% in other series [3, 7]. In addition to the AFC score, we also evaluated the prognostic value of a simple mCRS, derived from the traditional CRS established for patients with CRLM. Interestingly, this 4-point mCRS appears, in this series, to be of potentially higher prognostic value as compared with the AFC score. In univariate analysis, mCRS was predictive for OS but not for DFS. Even though mCRS was not prognostic in multivariate analysis and this very preliminary observation has to yet be confirmed in larger groups, it may suggest that similar pathways, eventually reflected by similar surrogates, could be responsible for tumor aggressiveness in CRLM and NCRNNELM. Along this line, even if the numbers in each category are limited, we did not observe a prognostic impact of the origin of the primary tumor, such as breast versus non-breast or digestive versus non-digestive. Also, this observation may potentially encourage others to test candidate biomarkers that have been recently demonstrated in CRLM in patients with NCRNNELM [20–26].

Based on our initial definition of the ER and LTS groups, with a postoperative DFS cut-off of ≤ 1 year and > 5 years, respectively, we observed that approximately, a quarter of the patients underwent oncologically futile surgery, whereas surgery was associated with a strong oncological benefit in less than 20% of the cases, underlining the lack of accuracy of current selection processes. Among baseline characteristics of primary tumors and liver metastases, none appeared to be reliable for distinguishing these two groups. Only a positive nodal status of the primary tumor and the size of liver metastases when they were diagnosed were different between these groups. However, due to large overlaps, these factors do not appear as potentially usable exclusion criteria for surgery. Furthermore, neither the AFC score nor the mCRS was found to discriminate between LTS and ER patients. When we compared the ER group with the patients with a DFS > 3 years, in whom a plausible surgical benefit could have been obtained, the rate of a positive nodal status of the primary tumor and the number of liver metastases at preoperative staging were significantly increased in the ER group. Interestingly, although the AFC score was not discriminating between these groups, the mCRS was significantly increased in ER patients as compared with the patients with a DFS > 3 years.

This work had several limitations. Mainly, this is a limited retrospective series, including highly selected patients. In addition, similarly to most of the studies in patients with NCRNNELM [2, 3, 7, 27, 28], the present series included a majority of patients with breast cancer liver metastases. However, no significant difference was observed when we compared postoperative outcomes in patients with breast and non-breast NCRNNELM.

In conclusion, although our overall results confirm that surgery could be effective in some patients with NCRNNELM, they also highlight the lack of accurate selection criteria for personalized therapeutic decision making. In this series, the previously described AFC score was unable to preoperatively identify the individual patients who would benefit from surgery. At the same time, a simple clinical score adapted from a prognostic model in CRLM showed some promising prognostic value, suggesting that similar biomarkers could be relevant in liver metastases, irrespective of the primary tumor origin. Taken together, these results underline the need for translational research to identify new biomarkers of individual tumor behavior in patients with NCRNNELM.

## Data Availability

The datasets used and/or analyzed during the current study are available from the corresponding author upon reasonable request.

## References

[1] Takemura N, Saiura A. Role of surgical resection for non-colorectal non-neuroendocrine liver metastases. World J Hepatol [Internet] 2017 [cited 2019 May 8];9(5):242–51. Available from: http://www.wjgnet.com/1948-5182/full/v9/i5/242.htm. 10.4254/wjh.v9.i5.242 PMC5316844 28261381

[2] Adam R, Chiche L, Aloia T, Elias D, Salmon R, Rivoire M, et al. Hepatic resection for noncolorectal nonendocrine liver metastases: analysis of 1,452 patients and development of a prognostic model. Ann Surg [Internet] 2006 [cited 2019 May 8];244(4):524–35. Available from: https://insights.ovid.com/crossref?an = 00153307-200601240-00021. 10.1097/01.sla.0000239036.46827.5f PMC1856551 16998361

[3] Hoffmann K, Bulut S, Tekbas A, Hinz U, Büchler MW, Schemmer P. Is hepatic resection for non-colorectal, non-neuroendocrine liver metastases justified? Ann Surg Oncol [Internet] 2015 [cited 2019 May 8];22 Suppl 3(S3):S1083-S1092. Available from: http://link.springer.com/10.1245/s10434-015-4775-x. 10.1245/s10434-015-4775-x 26242369

[4] Tan MCB, Jarnagin WR. Surgical management of non-colorectal hepatic metastasis. J Surg Oncol [Internet] 2014 [cited 2019 May 20];109(1):8–13. Available from: http://www.ncbi.nlm.nih.gov/pubmed/24122371. 10.1002/jso.23462 24122371

[5] Fitzgerald Timothy L., Brinkley Jason, Banks Shannon, Vohra Nasreen, Englert Zachary P., Zervos Emmanuel E. (2014). The benefits of liver resection for non-colorectal, non-neuroendocrine liver metastases: a systematic review. Langenbeck's Archives of Surgery.

[6] Schiergens TS, Lüning J, Renz BW, Thomas M, Pratschke S, Feng H, et al. Liver resection for non-colorectal non-neuroendocrine metastases: where do we stand today compared to colorectal cancer? J Gastrointest Surg [Internet] 2016 [cited 2019 May 8];20(6):1163–72. Available from: http://www.ncbi.nlm.nih.gov/pubmed/26921025. 10.1007/s11605-016-3115-1 26921025

[7] Lendoire J, Moro M, Andriani O, Grondona J, Gil O, Raffin G, et al. Liver resection for non-colorectal, non-neuroendocrine metastases: analysis of a multicenter study from Argentina. HPB (Oxford) [Internet] 2007 [cited 2019 May 8];9(6):435–9. Available from: http://www.ncbi.nlm.nih.gov/pubmed/18345290. 10.1080/13651820701769701 PMC2215356 18345290

[8] Tsang ME, Mahar AL, Martel G, Cleary SP, Nanji S, Ouellet J-F, et al. Assessing tools for management of noncolorectal nonneuroendocrine liver metastases: external validation of a prognostic model. J Surg Oncol [Internet] 2018 [cited 2019 May 20];118(6):1006–11. Available from: http://doi.wiley.com/10.1002/jso.25228. 10.1002/jso.25228 30196563

[9] Sim DPY, Goh BKP, Lee S-Y, Chan C-Y, Tan IBH, Cheow P-C, et al. Preoperative prognostic factors after liver resection for non-colorectal, non-neuroendocrine liver metastases and validation of the Adam score in an Asian Population. World J Surg [Internet] 2018 [cited 2019 May 20];42(4):1073–84. Available from: http://link.springer.com/10.1007/s00268-017-4208-z. 10.1007/s00268-017-4208-z 28875334

[10] Yedibela S, Gohl J, Graz V, Pfaffenberger MK, Merkel S, Hohenberger W, et al. Changes in indication and results after resection of hepatic metastases from noncolorectal primary tumors: a single-institutional review. Ann Surg Oncol [Internet] 2005 [cited 2019 May 8];12(10):778–85. Available from: http://www.springerlink.com/index/10.1245/ASO.2005.11.018. 10.1245/ASO.2005.11.018 16132374

[11] Fong Y, Fortner J, Sun RL, Brennan MF, Blumgart LH. Clinical score for predicting recurrence after hepatic resection for metastatic colorectal cancer: analysis of 1001 consecutive cases. Ann Surg [Internet] 1999 [cited 2019 May 8];230(3):309–18; discussion 318-21. Available from: http://www.ncbi.nlm.nih.gov/pubmed/10493478. 10.1097/00000658-199909000-00004 PMC1420876 10493478

[12] Therasse P, Arbuck SG, Eisenhauer EA, Wanders J, Kaplan RS, Rubinstein L, et al. New guidelines to evaluate the response to treatment in solid tumors. European Organization for Research and Treatment of Cancer, National Cancer Institute of the United States, National Cancer Institute of Canada. J Natl Cancer Inst [Internet] 2000 [cited 2020 Feb 1];92(3):205–16. Available from: http://www.ncbi.nlm.nih.gov/pubmed/10655437. 10.1093/jnci/92.3.205 10655437

[13] Dindo D, Demartines N, Clavien P-A. Classification of surgical complications: a new proposal with evaluation in a cohort of 6336 patients and results of a survey. Ann Surg [Internet] 2004 [cited 2019 May 8];240(2):205–13. Available from: http://www.ncbi.nlm.nih.gov/pubmed/15273542. 10.1097/01.sla.0000133083.54934.ae PMC1360123 15273542

[14] Van Cutsem E, Cervantes A, Adam R, Sobrero A, Van Krieken JH, Aderka D, et al. ESMO consensus guidelines for the management of patients with metastatic colorectal cancer. Ann Oncol [Internet] 2016 [cited 2019 May 8];27(8):1386–422. Available from: https://academic.oup.com/annonc/article-lookup/doi/10.1093/annonc/mdw235. 10.1093/annonc/mdw235 27380959

[15] Hellman S, Weichselbaum RR. Oligometastases. J Clin Oncol [Internet] 1995 [cited 2019 May 8];13(1):8–10. Available from: http://ascopubs.org/doi/10.1200/JCO.1995.13.1.8. 10.1200/JCO.1995.13.1.8 7799047

[16] Weichselbaum RR, Hellman S. Oligometastases revisited. Nat Rev Clin Oncol [Internet] 2011 [cited 2019 May 8];8(6):378–82. Available from: http://www.nature.com/articles/nrclinonc.2011.44. 10.1038/nrclinonc.2011.44 21423255

[17] Guckenberger M, Lievens Y, Bouma AB, Collette L, Dekker A (2020). deSouza NM, et al. Characterisation and classification of oligometastatic disease: a European Society for Radiotherapy and Oncology and European Organisation for Research and Treatment of Cancer consensus recommendation. Lancet Oncol..

[18] Groeschl RT, Nachmany I, Steel JL, Reddy SK, Glazer ES, de Jong MC, et al. Hepatectomy for noncolorectal non-neuroendocrine metastatic cancer: a multi-institutional analysis. J Am Coll Surg [Internet] 2012 [cited 2019 May 8];214(5):769–77. Available from: https://linkinghub.elsevier.com/retrieve/pii/S1072751512000865. 10.1016/j.jamcollsurg.2011.12.048 22425166

[19] O’Rourke TR, Tekkis P, Yeung S, Fawcett J, Lynch S, Strong R, et al. Long-term results of liver resection for non-colorectal, non-neuroendocrine metastases. Ann Surg Oncol [Internet] 2008 [cited 2019 May 8];15(1):207–18. Available from: http://www.springerlink.com/index/10.1245/s10434-007-9649-4. 10.1245/s10434-007-9649-4 17963007

[20] Eefsen RL, Vermeulen PB, Christensen IJ, Laerum OD, Mogensen MB, Rolff HC, et al. Growth pattern of colorectal liver metastasis as a marker of recurrence risk. Clin Exp Metastasis [Internet] 2015 [cited 2019 May 20];32(4):369–81. Available from: http://link.springer.com/10.1007/s10585-015-9715-4. 10.1007/s10585-015-9715-4 25822899

[21] Nierop PMH, Galjart B, Höppener DJ, van der Stok EP, Coebergh van den Braak RRJ, Vermeulen PB, et al. Salvage treatment for recurrences after first resection of colorectal liver metastases: the impact of histopathological growth patterns. Clin Exp Metastasis [Internet] 2019 [cited 2019 Aug 2];36(2):109–18. Available from: http://www.ncbi.nlm.nih.gov/pubmed/30843120. 10.1007/s10585-019-09960-7 PMC6445820 30843120

[22] Fridman Wolf Herman, Pagès Franck, Sautès-Fridman Catherine, Galon Jérôme (2012). The immune contexture in human tumours: impact on clinical outcome. Nature Reviews Cancer.

[23] Pagès F, Mlecnik B, Marliot F, Bindea G, Ou F-S, Bifulco C, et al. International validation of the consensus immunoscore for the classification of colon cancer: a prognostic and accuracy study. Lancet [Internet] 2018 [cited 2019 Oct 6];391(10135):2128–39. Available from: http://www.ncbi.nlm.nih.gov/pubmed/29754777. 10.1016/S0140-6736(18)30789-X 29754777

[24] Galon J. (2006). Type, Density, and Location of Immune Cells Within Human Colorectal Tumors Predict Clinical Outcome. Science.

[25] Pitroda SP, Khodarev NN, Huang L, Uppal A, Wightman SC, Ganai S, et al. Integrated molecular subtyping defines a curable oligometastatic state in colorectal liver metastasis. Nat Commun [Internet] 2018 [cited 2019 Oct 6];9(1):1793. Available from: http://www.nature.com/articles/s41467-018-04278-6. 10.1038/s41467-018-04278-6 PMC5935683 29728604

[26] Pitroda SP, Khodarev NN, Huang L, Uppal A, Wightman SC, Ganai S, et al. Integrated molecular subtyping defines a curable oligometastatic state in colorectal liver metastasis. Nat Commun [Internet] 2018 [cited 2019 May 20];9(1):1793. Available from: http://www.ncbi.nlm.nih.gov/pubmed/29728604. 10.1038/s41467-018-04278-6 PMC5935683 29728604

[27] Ercolani G, Grazi GL, Ravaioli M, Ramacciato G, Cescon M, Varotti G, et al. The role of liver resections for noncolorectal, nonneuroendocrine metastases: experience with 142 observed cases. Ann Surg Oncol [Internet] 2005 [cited 2019 May 20];12(6):459–66. Available from: http://www.ncbi.nlm.nih.gov/pubmed/15886903. 10.1245/ASO.2005.06.034 15886903

[28] Karavias D.D., Tepetes K., Karatzas T., Felekouras E., Androulakis J. (2002). Liver resection for metastatic non-colorectal non-neuroendocrine hepatic neoplasms. European Journal of Surgical Oncology (EJSO).

